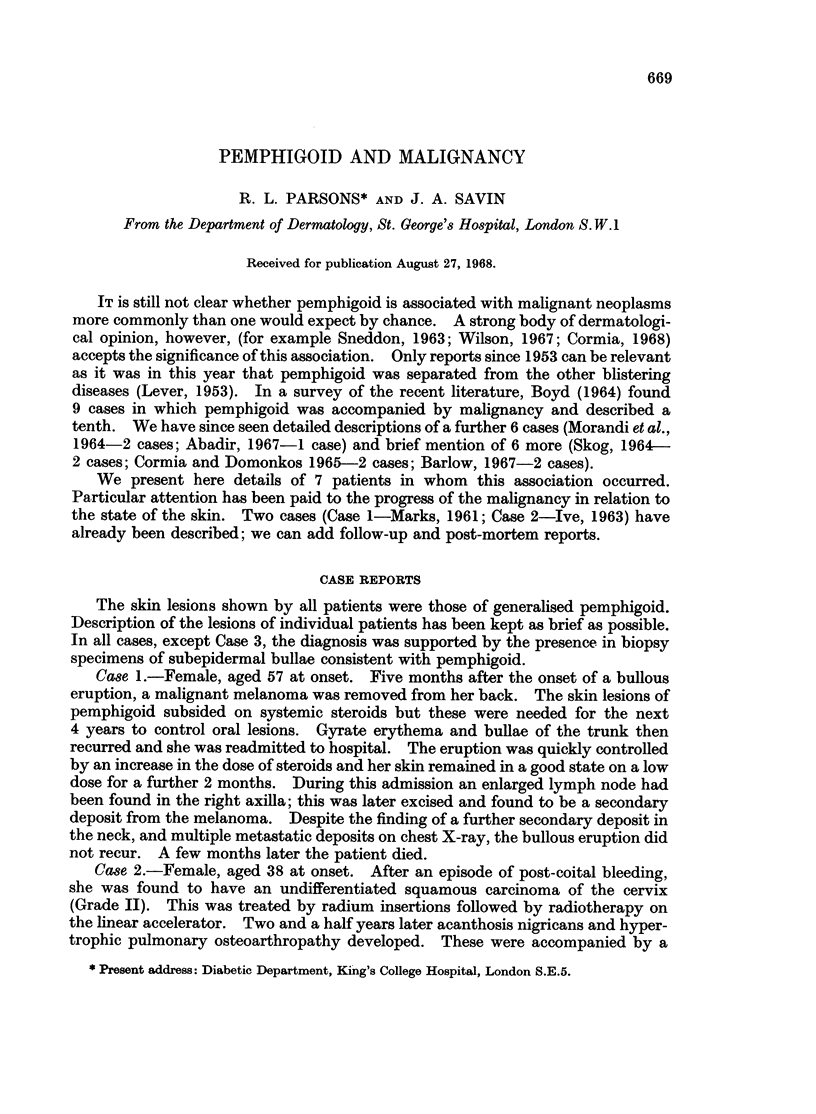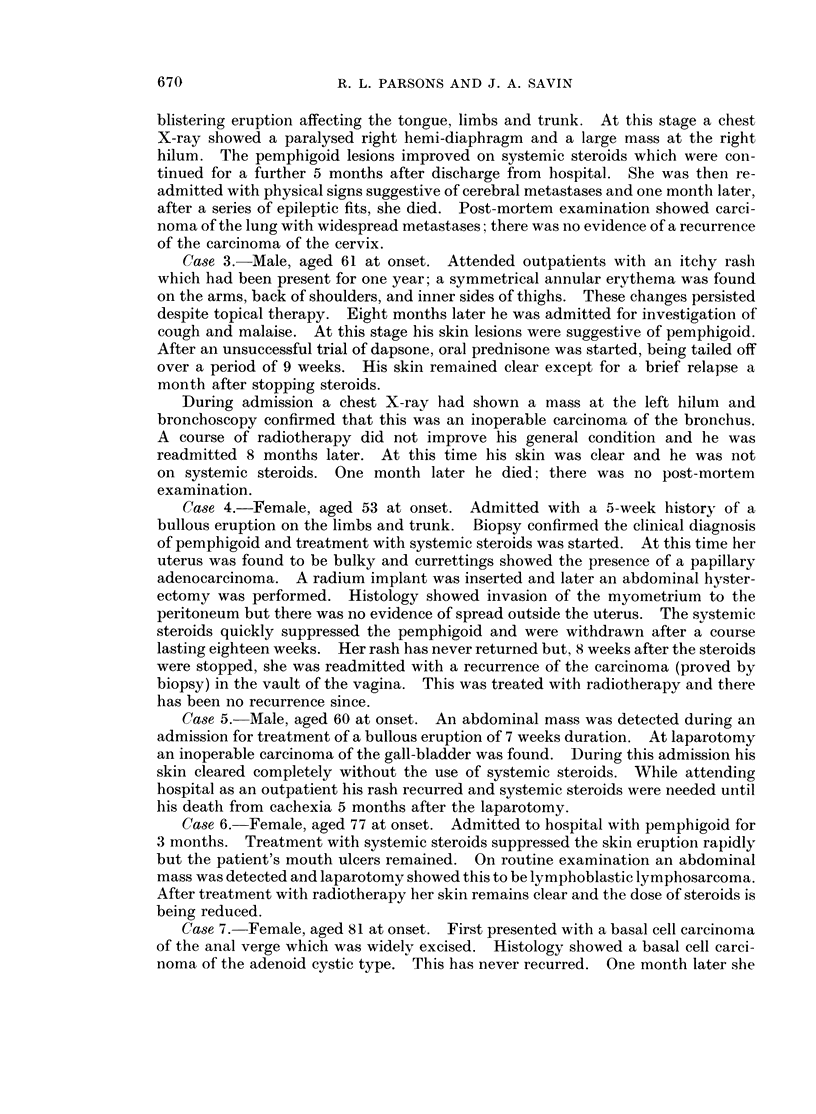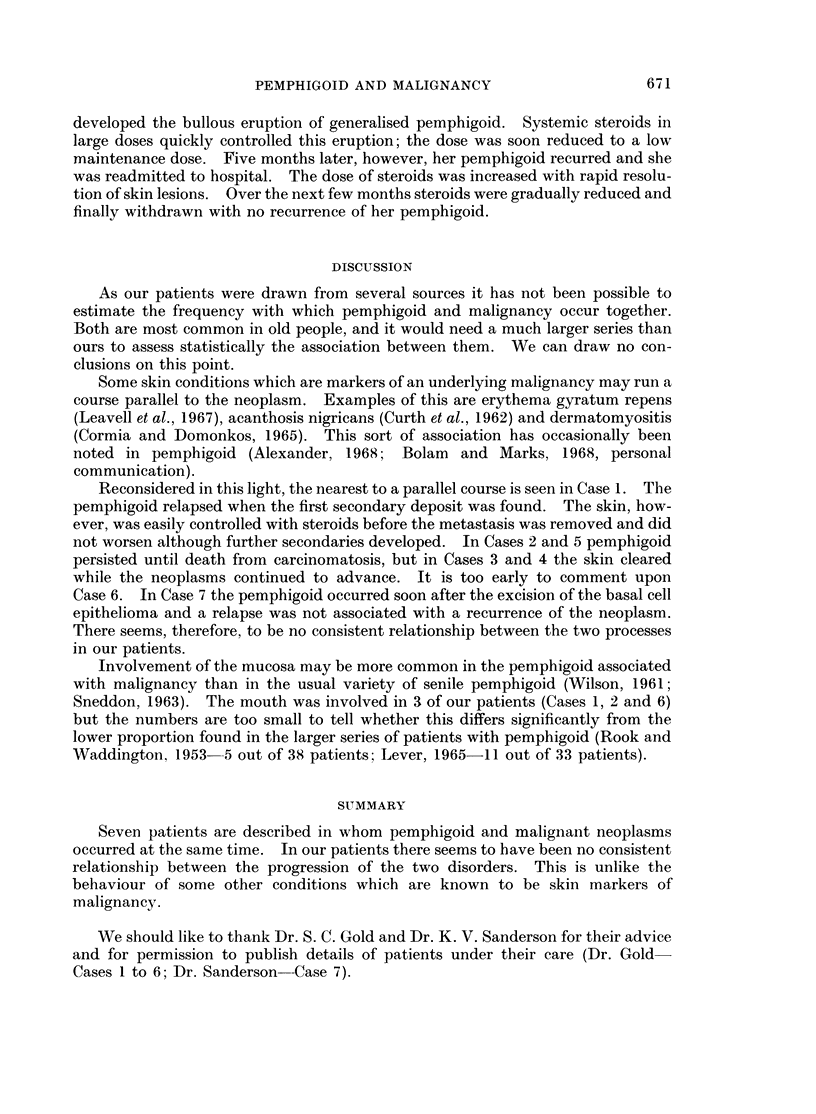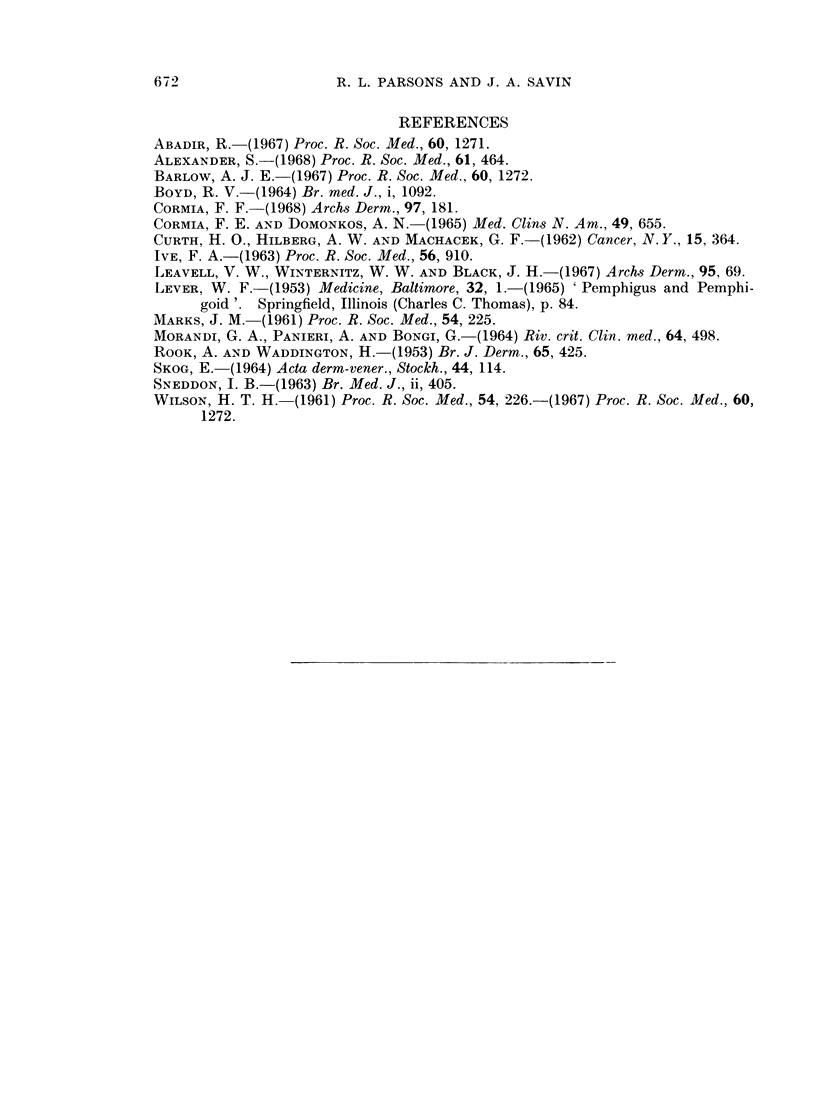# Pemphigoid and malignancy.

**DOI:** 10.1038/bjc.1968.79

**Published:** 1968-12

**Authors:** R. L. Parsons, J. A. Savin


### PEMPHIGOID AND MALIGNANCY

R. L. PARSONS* AND J. A. SAVIN

*From the Department of Dermatology, St. George's Hospital, London S.W.1*

Received for publication August 27, 1968.

IT is still not clear whether pemphigoid is associated with malignant neoplasms more commonly than one would expect by chance. A strong body of dermatological opinion, however, (for example Sneddon, 1963; Wilson, 1967; Cormia, 1968) accepts the significance of this association. Only reports since 1953 can be relevant as it was in this year that pemphigoid was separated from the other blistering diseases (Lever, 1953). In a survey of the recent literature, Boyd (1964) found 9 cases in which pemphigoid was accompanied by malignancy and described a tenth. We have since seen detailed descriptions of a further 6 cases (Morandi *et al.*, 1964—2 cases; Abadir, 1967—1 case) and brief mention of 6 more (Skog, 1964—2 cases; Cormia and Domonkos 1965—2 cases; Barlow, 1967—2 cases).

We present here details of 7 patients in whom this association occurred. Particular attention has been paid to the progress of the malignancy in relation to the state of the skin. Two cases (Case 1—Marks, 1961; Case 2—Ive, 1963) have already been described; we can add follow-up and post-mortem reports.

### CASE REPORTS

The skin lesions shown by all patients were those of generalised pemphigoid. Description of the lesions of individual patients has been kept as brief as possible. In all cases, except Case 3, the diagnosis was supported by the presence in biopsy specimens of subepidermal bullae consistent with pemphigoid.

*Case* 1.—Female, aged 57 at onset. Five months after the onset of a bullous eruption, a malignant melanoma was removed from her back. The skin lesions of pemphigoid subsided on systemic steroids but these were needed for the next 4 years to control oral lesions. Gyrate erythema and bullae of the trunk then recurred and she was readmitted to hospital. The eruption was quickly controlled by an increase in the dose of steroids and her skin remained in a good state on a low dose for a further 2 months. During this admission an enlarged lymph node had been found in the right axilla; this was later excised and found to be a secondary deposit from the melanoma. Despite the finding of a further secondary deposit in the neck, and multiple metastatic deposits on chest X-ray, the bullous eruption did not recur. A few months later the patient died.

*Case* 2.—Female, aged 38 at onset. After an episode of post-coital bleeding, she was found to have an undifferentiated squamous carcinoma of the cervix (Grade II). This was treated by radium insertions followed by radiotherapy on the linear accelerator. Two and a half years later acanthosis nigricans and hypertrophic pulmonary osteoarthropathy developed. These were accompanied by a

* Present address: Diabetic Department, King's College Hospital, London S.E.5.

blistering eruption affecting the tongue, limbs and trunk. At this stage a chest X-ray showed a paralysed right hemi-diaphragm and a large mass at the right hilum. The pemphigoid lesions improved on systemic steroids which were continued for a further 5 months after discharge from hospital. She was then readmitted with physical signs suggestive of cerebral metastases and one month later, after a series of epileptic fits, she died. Post-mortem examination showed carcinoma of the lung with widespread metastases; there was no evidence of a recurrence of the carcinoma of the cervix.

*Case* 3.—Male, aged 61 at onset. Attended outpatients with an itchy rash which had been present for one year; a symmetrical annular erythema was found on the arms, back of shoulders, and inner sides of thighs. These changes persisted despite topical therapy. Eight months later he was admitted for investigation of cough and malaise. At this stage his skin lesions were suggestive of pemphigoid. After an unsuccessful trial of dapsone, oral prednisone was started, being tailed off over a period of 9 weeks. His skin remained clear except for a brief relapse a month after stopping steroids.

During admission a chest X-ray had shown a mass at the left hilum and bronchoscopy confirmed that this was an inoperable carcinoma of the bronchus. A course of radiotherapy did not improve his general condition and he was readmitted 8 months later. At this time his skin was clear and he was not on systemic steroids. One month later he died; there was no post-mortem examination.

*Case* 4.—Female, aged 53 at onset. Admitted with a 5-week history of a bullous eruption on the limbs and trunk. Biopsy confirmed the clinical diagnosis of pemphigoid and treatment with systemic steroids was started. At this time her uterus was found to be bulky and currettings showed the presence of a papillary adenocarcinoma. A radium implant was inserted and later an abdominal hysterectomy was performed. Histology showed invasion of the myometrium to the peritoneum but there was no evidence of spread outside the uterus. The systemic steroids quickly suppressed the pemphigoid and were withdrawn after a course lasting eighteen weeks. Her rash has never returned but, 8 weeks after the steroids were stopped, she was readmitted with a recurrence of the carcinoma (proved by biopsy) in the vault of the vagina. This was treated with radiotherapy and there has been no recurrence since.

*Case* 5.—Male, aged 60 at onset. An abdominal mass was detected during an admission for treatment of a bullous eruption of 7 weeks duration. At laparotomy an inoperable carcinoma of the gall-bladder was found. During this admission his skin cleared completely without the use of systemic steroids. While attending hospital as an outpatient his rash recurred and systemic steroids were needed until his death from cachexia 5 months after the laparotomy.

*Case* 6.—Female, aged 77 at onset. Admitted to hospital with pemphigoid for 3 months. Treatment with systemic steroids suppressed the skin eruption rapidly but the patient's mouth ulcers remained. On routine examination an abdominal mass was detected and laparotomy showed this to be lymphoblastic lymphosarcoma. After treatment with radiotherapy her skin remains clear and the dose of steroids is being reduced.

*Case* 7.—Female, aged 81 at onset. First presented with a basal cell carcinoma of the anal verge which was widely excised. Histology showed a basal cell carcinoma of the adenoid cystic type. This has never recurred. One month later she developed the bullous eruption of generalised pemphigoid. Systemic steroids in large doses quickly controlled this eruption; the dose was soon reduced to a low maintenance dose. Five months later, however, her pemphigoid recurred and she was readmitted to hospital. The dose of steroids was increased with rapid resolution of skin lesions. Over the next few months steroids were gradually reduced and finally withdrawn with no recurrence of her pemphigoid.

### DISCUSSION

As our patients were drawn from several sources it has not been possible to estimate the frequency with which pemphigoid and malignancy occur together. Both are most common in old people, and it would need a much larger series than ours to assess statistically the association between them. We can draw no conclusions on this point.

Some skin conditions which are markers of an underlying malignancy may run a course parallel to the neoplasm. Examples of this are erythema gyratum repens (Leavell *et al.*, 1967), acanthosis nigricans (Curth *et al.*, 1962) and dermatomyositis (Cormia and Domonkos, 1965). This sort of association has occasionally been noted in pemphigoid (Alexander, 1968; Bolam and Marks, 1968, personal communication).

Reconsidered in this light, the nearest to a parallel course is seen in Case 1. The pemphigoid relapsed when the first secondary deposit was found. The skin, however, was easily controlled with steroids before the metastasis was removed and did not worsen although further secondaries developed. In Cases 2 and 5 pemphigoid persisted until death from carcinomatosis, but in Cases 3 and 4 the skin cleared while the neoplasms continued to advance. It is too early to comment upon Case 6. In Case 7 the pemphigoid occurred soon after the excision of the basal cell epithelioma and a relapse was not associated with a recurrence of the neoplasm. There seems, therefore, to be no consistent relationship between the two processes in our patients.

Involvement of the mucosa may be more common in the pemphigoid associated with malignancy than in the usual variety of senile pemphigoid (Wilson, 1961; Sneddon, 1963). The mouth was involved in 3 of our patients (Cases 1, 2 and 6) but the numbers are too small to tell whether this differs significantly from the lower proportion found in the larger series of patients with pemphigoid (Rook and Waddington, 1953—5 out of 38 patients; Lever, 1965—11 out of 33 patients).

### SUMMARY

Seven patients are described in whom pemphigoid and malignant neoplasms occurred at the same time. In our patients there seems to have been no consistent relationship between the progression of the two disorders. This is unlike the behaviour of some other conditions which are known to be skin markers of malignancy.

We should like to thank Dr. S. C. Gold and Dr. K. V. Sanderson for their advice and for permission to publish details of patients under their care (Dr. Gold—Cases 1 to 6; Dr. Sanderson—Case 7).

### REFERENCES

Abadir, R.—(1967) *Proc. R. Soc. Med.*, **60**, 1271.
Alexander, S.—(1968) *Proc. R. Soc. Med.*, **61**, 464.
Barlow, A. J. E.—(1967) *Proc. R. Soc. Med.*, **60**, 1272.
Boyd, R. V.—(1964) *Br. med. J.*, i, 1092.
Cormia, F. F.—(1968) *Archs Derm.*, **97**, 181.
Cormia, F. E. and Domonkos, A. N.—(1965) *Med. Clins N. Am.*, **49**, 655.
Curth, H. O., Hilberg, A. W. and Machacek, G. F.—(1962) *Cancer, N.Y.*, **15**, 364.
Ive, F. A.—(1963) *Proc. R. Soc. Med.*, **56**, 910.
Leavell, V. W., Winternitz, W. W. and Black, J. H.—(1967) *Archs Derm.*, **95**, 69.
Lever, W. F.—(1953) *Medicine, Baltimore*, **32**, 1.—(1965) ‘ Pemphigus and Pemphigoid ’. Springfield, Illinois (Charles C. Thomas), p. 84.
Marks, J. M.—(1961) *Proc. R. Soc. Med.*, **54**, 225.
Morandi, G. A., Panieri, A. and Bongi, G.—(1964) *Riv. crit. Clin. med.*, **64**, 498.
Rook, A. and Waddington, H.—(1953) *Br. J. Derm.*, **65**, 425.
Skog, E.—(1964) *Acta derm-vener., Stockh.*, **44**, 114.
Sneddon, I. B.—(1963) *Br. Med. J.*, ii, 405.
Wilson, H. T. H.—(1961) *Proc. R. Soc. Med.*, **54**, 226.—(1967) *Proc. R. Soc. Med.*, **60**, 1272.